# Influence of a day hospice on the quality of life of palliative care patients: an interview-based qualitative survey

**DOI:** 10.1186/s12904-026-02052-w

**Published:** 2026-03-11

**Authors:** Ulrich Kaiser, Ursula Vehling-Kaiser, Felix Kaiser, Ana Hoffmann, Eleene Kaiser, Moritz Fiedler, Michael Rechenmacher, Florian Kaiser

**Affiliations:** 1 Clinic and Polyclinic for Internal Medicine III, University Hospital Regensburg, Franz-Josef-Strauß-Allee 11, Regensburg, 93053 Germany; 2 Day Hospice Adiuvantes, Vilsbiburg, Germany; 3 MVZ Dr. Vehling-Kaiser GmbH, Landshut, Germany; 4 VK&K Studien GbR, Landshut, Germany; 5 Center for Palliative Medicine, University Hospital Regensburg, Regensburg, Germany

**Keywords:** Palliative medicine, Day hospice, Palliative patients, Quality of life, Palliative care

## Abstract

**Background:**

In addition to the established forms of outpatient palliative care, day hospices have been available in Germany as a further component of care for several years. However, only a few day hospices currently exist in Germany, and data on the effects of day hospices, particularly on the quality of life and care situation of the patients concerned, are not sufficient. Therefore, this study aimed to investigate the influence of day hospice care on the quality of life of palliative care patients.

**Methods:**

This qualitative interview study included palliative care patients at a day hospice in Lower Bavaria. In collaboration with SINUS Markt- und Sozialforschung GmbH, Heidelberg, qualitative guided interviews were conducted between October and December 2024. The interview guide was developed by the team of authors in collaboration with SINUS GmbH and was agreed upon after several rounds of discussions. Interviews were recorded, transcribed verbatim, and anonymized. The transcripts were evaluated using hermeneutic text interpretation.

**Results:**

A total of 20 palliative care patients (13 females and 7 males) with an average age of 70.9 years (range, 56–90 years) participated in the study. All participants had a malignant disease. Participants reported that visiting the day hospice improved their quality of life. They reported that social factors were more important than medical factors in improving quality of life. The aspects of community, professional support and guidance, and relief for relatives were cited as particularly important.

**Conclusions:**

The participants perceived the day hospice as a place of peace and relaxation where they could regain strength and confidence and rediscover meaning and joy in their lives. Therefore, day hospices are a fundamentally valuable component of outpatient palliative care. The further development of this care structure will be closely watched with great interest.

**Trial registration:**

The study was registered as part of the IMPULS-Study in the German Register of Clinical Studies (DRKS-ID: DRKS00031613; Date of registration: 04 April 2023) and the Display portal of the Center for Clinical Studies of the University Hospital Regensburg (Z-2022-1734-6; Date of registration: 01 October 2022).

**Supplementary Information:**

The online version contains supplementary material available at 10.1186/s12904-026-02052-w.

## Background

With the advances in modern treatment modalities, palliative medicine has become increasingly important in the care of patients with advanced disease in both outpatient and inpatient settings [1]. In Germany, day hospices have been available as a component of care for several years, complementing established forms of outpatient palliative care, such as general and specialized outpatient palliative care (AAPV, SAPV) [2, 3].

Day hospices provide care for terminally ill adults and are open to all affected individuals regardless of their social background [1, 4–6]. In day hospices, patients are cared for by palliative care nurses during the day and then return to their home environment. Day hospice visits can occur on one or more days per week, provided certain conditions are met, including approval from the relevant health insurance provider and a general health state sufficient for active participation in the day program [2].

Day hospices provide opportunities for interaction with individuals facing similar circumstances, creative activities, and optimal palliative care, with the aim of improving quality of life, facilitating social reintegration of palliative care patients, and reducing the burden on family caregivers [4, 7].

Several international reviews have examined the effects of day hospices and provided initial indications of a high level of satisfaction among day hospice guests, with social support received, relaxing environment, and opportunities for interpersonal interaction being highlighted as key reasons [8, 9]. Although quantitative studies conducted to date have not reported any improvement in the overall quality of life, symptoms have been reduced in certain areas, and emotional well-being has been enhanced [10, 11].

The direct applicability of the findings of these international studies to the German healthcare system is limited due to differences in healthcare systems and the heterogeneity of day hospice structures in individual studies [1, 3].

In Germany, patients are referred to day hospices by various medical professionals from both outpatient and inpatient settings, including general practitioners, hematologists, oncologists, and palliative care specialists. In principle, however, any doctor can make a referral. Day hospices are open to patients with advanced, incurable illnesses. But, as is currently still common in palliative care, hematological and oncological patients represent the majority of day hospices guests. At the same time, however, patients must still be physically and mentally capable of integrating themselves into the community of a day hospice and participating in joint social activities. If a patient’s health deteriorates to the point where they are no longer able to attend the day hospice, further care arrangements such as 24-hour care, hospital, or hospice are planned in advance wherever possible. In principle, this also applies to patients in the pre-terminal and terminal stages, who usually no longer visit the day hospice. Instead, palliative care is provided in advance on an outpatient setting at home (e.g., specialized outpatient palliative care) or on an inpatient setting (e.g., hospice, hospital).

The day hospice participating in this study, organized as a non-profit company, has eight places with a total of five palliative care nurses. The therapeutic focus is on both palliative care and social aspects.

Only a few day hospices are currently available in Germany. Despite initial indications suggesting that day hospices may offer potential benefits [5], data on the effects of day hospices, particularly on the quality of life and care situation of the patients concerned, in Germany are limited. Therefore, this study aimed to examine the effects of day hospice care on patients’ quality of life.

## Methods

### Ethics

This study was conducted as part of the IMPULS study (investigation into the implementation, realization, and benefits of a day hospice, German Clinical Trials Register: DRKS-ID DRKS00031613, Center for Clinical Trials of the University Hospital Regensburg: Z-2022-1734-6 [12]), which was approved by the Ethics Committee of the University of Regensburg (Approval No. 22-3012_1-101). All approvals were granted in full compliance with the principles of the Declaration of Helsinki.

### Interview participants

This qualitative interview study included guests visiting a day hospice in Lower Bavaria between October and December 2024. Participants were recruited by the study team at VK&K Studien GbR. The inclusion criteria were sufficient knowledge of German, adequate physical condition, active use of the day hospice, and written consent to participate in the study. Written informed consent was obtained from all participants prior to their participation in the study, in accordance with ethical standards.

### Description of the day hospice

The day hospice is located in a rural area, was founded in 2022, and is open Monday through Friday from 8:00 am to 4:00 pm. In addition to social and office rooms, it has a living-dining kitchen, a lounge, two single rooms, a doctor’s office, and a large garden area. It provides nursing and supportive palliative care measures, weekly medical consultations, experience-oriented activities in the spirit of adventure therapy, and occupational therapy (e.g., aromatherapy and sports therapy). Care is provided by appropriately qualified palliative care nurses.

### Conducting interviews

In collaboration with SINUS Markt- und Sozialforschung GmbH, Heidelberg, qualitative guided interviews were conducted with the participants by telephone by employees of SINUS GmbH. Telephone interviews provided maximum flexibility in scheduling and implementation, and the interview guide ensured that all relevant aspects were addressed as far as possible (see Appendix).

**Fig. 1:**
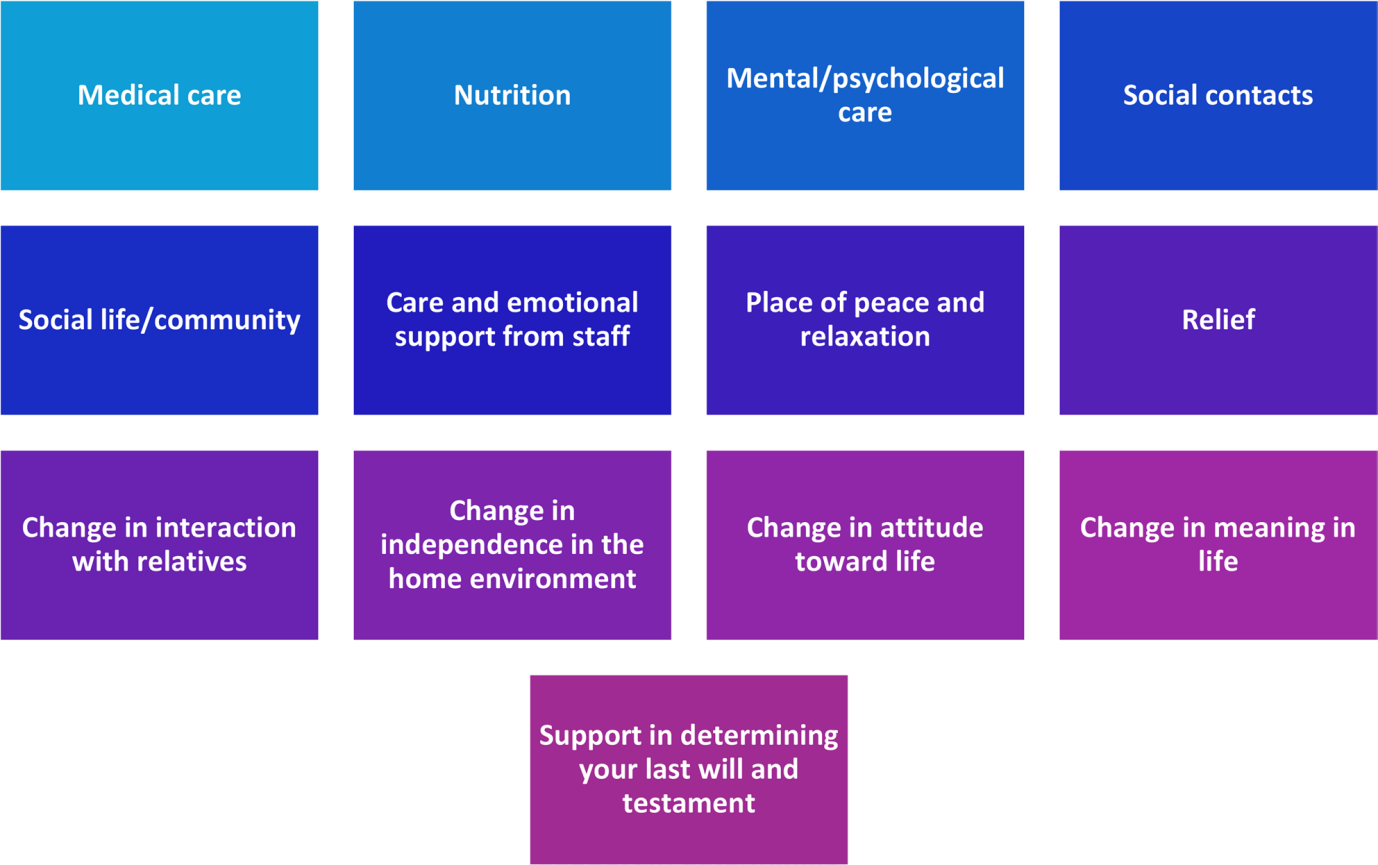
Key points of the interview guide used

The semistructured interview guide was developed by the authors in collaboration with SINUS GmbH, approved after several rounds of discussions, and subsequently validated for comprehensibility and clarity in a series of pretests. During the creation process, the topics specified and areas of interest identified by the study management were used for operationalization, with particular emphasis placed on designing questions as sensitively as possible to reduce the natural distance between researchers and study participants. This approach enables respondents to freely and openly express their views about what they consider important and worth mentioning in their natural everyday language [13–15]. Based on the interviewees’ statements, the interviewer asked in-depth questions. The interview guide focused on quality of life and reasons for potential changes in this area (Fig. 1 and Appendix).

### Evaluation of interviews

The interviews were recorded and then transcribed verbatim and anonymized. The interviews were evaluated using hermeneutic text interpretation as a common method of qualitative empirical social research, allowing for interpretation and structuring of the results according to connecting units of meaning [16]. The aim of the IMPULS study – in which this work is embedded – was, among other things, to conduct a global investigation of day hospices at various levels. The hermeneutic method offers the advantage of enabling a very deep and contextualised interpretation of data, taking into account multidimensional meanings. Day hospices have an impact not only in the medical-palliative context, but also in the social and even family environment. The data collected in the interviews is therefore complex and intersectoral. A flexible and reflexive approach, as enabled by the hermeneutic methodology, was therefore necessary for a comprehensive and in-depth interpretation and evaluation.

## Results

A total of 20 patients receiving palliative care at a day hospice in Lower Bavaria participated in the survey. Of the 20 participants, 13 were females, and 7 were males. Further information was provided for 14 participants (average age, 70.9 years; range, 56–90 years; ECOG performance status, 0: 8%, 1: 35%, 2: 40%, 3: 17%; housing situation: living alone: 43%, living with relatives: 57%; level of care: yes: 40%, no: 60%). The average length of stay in the day hospice at the time of the survey was 37 days (range 7 days − 252 days). All participants had a malignant hematological or oncological disease at an advanced stage, either under a best supportive care concept or under the last available lines of therapy.

The average duration of the interviews was 26 min (range: 5–50 min). The duration varied based on the form of the respondents on the day, their resilience, and their availability. The participants were highly engaged, interested, and willing to provide information to the best of their ability during the interview. All participants were able to adequately respond to the questions asked.

The interviews were evaluated, and the corresponding results were presented. Quotations were linked to the respective interview participants by assigning a number to each.

### Overall quality of life

The participants reported that visiting the day hospice had a positive impact on their quality of life. They emphasized that the visits were crucial to them, and they eagerly anticipated the days at the day hospice during the week and were sad when they were over.

Quote (9): “*Well, I’m always sad when the day is over.… When the taxi arrives to pick me up, I think, ´Oh, it’s over, what a shame*´.”

The participants reported that new social contacts, a newfound zest for life, and comprehensive care provided by the staff were the reasons for the improved quality of life. Many participants expressed, *“…that this is a very important institution that should exist more often”* (5).

### Specific factors in improving quality of life

The reasons for the perceived improvement in quality of life through the day hospice varied in importance. Social and nursing factors were mentioned more frequently than medical factors. The day hospice was perceived as *“…a place in the normal world… where I can get myself sorted out and live a normal life again”* (15).

### Medical care

Many participants reported that the medical care provided at the day hospice is important for improving their quality of life. The day hospice offers treatment measures, such as regular physician visits, blood sampling, infusions, parenteral nutrition, and changing a pain pump. Therefore, visits to the physician are avoided and *“…you don’t always have to sit around in a waiting room somewhere…”* (10). Additionally, medical care is provided in a family setting, which was a positive aspect.

However, the participants reported that the approval process of health insurance companies was problematic. The uncertainty about the finally approved length of stay is a critical aspect that patients find stressful.

Quote (5): *“I’ll have to wait and see what happens with the extension. But you hear from others too that it’s often a struggle.”*

### Nutrition

Shared meals promoted a sense of community and social contact, thereby contributing to well-being because it is *“nicer in the community than when I’m served food at home and told that I absolutely have to eat”* (14). Although some patients were well cared for at home, regular meals contributed to improved quality of life for others because this option was not always available at home.

Quote (20): *“Thanks to the day hospice*,* I at least eat*,* which is a good start. At home*,* I’m sometimes too tired and just don’t bother.”*

Parenteral nutrition was important for some participants and was mentioned as a factor in improving quality of life. The consideration given to the individual wishes and needs of each patient regarding food (e.g., portion size) was also highlighted as a positive aspect.

Quote (14): *“And they make sure… that I can eat my food and drink plenty of fluids… but without any pressure from my relatives….”*

### Mental/psychological care

Some participants reported that the availability of psychological support at the day hospice was a key factor in improving their quality of life, and this support would not have been available to them without the day hospice. This form of support in a day hospice is significantly different from a hospital stay.

Quote (16): *“…in the hospital*,* you are basically put back together again… but it’s this intermediate stage*,* the human aspect*,* that is often sorely lacking….”*

Communication with the nursing staff is particularly appreciated. However, communication with fellow patients and pastoral caregivers is also important. The participants described both communication and the overall experience of visiting the day hospice as psychologically relieving, as *“the soul is really emphasized here”* (14).

### Social contacts

Most participants cited social interaction at the day hospice as the most important factor in improving their quality of life, as this counteracts isolation and loneliness.

Quote (13): *“…I live alone in an apartment building where people don’t even greet each other*,* so you can’t make any friends. It’s wonderful there now.”*

Conversely, some participants reported that social contact was not their primary goal in visiting the day hospice, as some were generally less interested in social contact, whereas others were primarily looking for a place to rest. Participants who perceived a lack of social contact seeking in others often found this rather strange. However, they tolerated the situation.

For most participants, the day hospice was a place where they could maintain social contacts and occasionally develop closer relationships that extended beyond the scope of their visits. For some guests, the constant comings and goings of different patients were an obstacle to forming a closer community. However, others saw this as an opportunity to exchange ideas with as many different conversation partners as possible.

Despite the associated stress, most participants had come to terms with the death of fellow patients and considered death natural and inevitable in relation to their situation. Additionally, their quality of life was not affected to such an extent that they stopped visiting the day hospice.

Quote (2): *“…I don’t really see it as a bad thing…. Others say*,* ‘Oh*,* day hospice…. What’s that? Don’t go there….’ And I… say*,* ‘Okay. It’s good for you*,* so I’ll do it.’”*.

The aspect of “mental strengthening through social contact” is highly valued. According to the participants, this aspect improved their quality of life, as the day hospice could compensate for social contacts that might have been significantly reduced in the patients’ everyday lives. Interaction can alleviate loneliness, take your mind off things, and improve well-being. Additionally, participants reported that the exchange helped them cope better with their illness, whereas others particularly appreciated the distraction provided by the day hospice.

Quote (9): *“So the fewer contacts you have*,* which adds to the illness*,* the more isolated you become. Or you feel much worse. I really enjoy being here….”*

Many patients reported that the feeling of lightheartedness and normality that arises in the day hospice was essential for their well-being. All participants shared the same fate. Therefore, topics such as cancer need not be explained in detail. Because *“…often when you have cancer*,* people don’t dare to call you anymore. Or you become defined by your cancer. That’s not the case here.… That’s what makes it special*,* I think. Being able to live a simple life again”* (15).

### Social life/community

Variety, fun, and communication improved the attitude of many patients toward life and quality of life.

Quote (15): *“…I was pleasantly surprised at how quickly I started laughing again. And you just feel better again.”*

Most participants particularly appreciated the sense of community at the day hospice, where they could enjoy pleasant experiences and have fun together, rather than focusing solely on the negative aspects. The importance of this aspect was particularly emphasized by participants who had little interaction at home.

Quote (4): *“I’m having fun again. That I can talk to someone again. Something I don’t have all week. Yes. It’s*,* how can I put it*,* it makes me happy.”*

At the day hospice, guests are offered variety and distraction, with some becoming very actively involved in shaping social life at the day hospice.

For many participants, social interaction at the day hospice is a substitute for lost or reduced social activities and is essential for maintaining or improving their quality of life. Only a few patients were socially integrated in their everyday lives at home to the extent that they were less dependent on social life at the day hospice.

Quote (15): *“…and when you’re here all day*,* it really feels like nighttime [in terms of how you feel]*,* like when you used to work all day. Because you have conversations*,* and then you do something else….”*

Participants appreciated the communal meals, the varied food, and the opportunity to cook with others, which contributed to improving their quality of life. However, the feeling of community always took priority, even when the illness meant that little or only specific food could be consumed.

Many participants emphasized that the day hospice gave them the opportunity to participate in activities they would not otherwise be able to do.

Quote (9): *“My children wouldn’t have dared to do that with me alone. We also do a lot of things here that we just enjoy.”*

Participants reported that this contributed to improving their quality of life. They appreciated the diverse program, particularly pedicures, massage, and visits to the hairdresser, which gave them a sense of self-awareness and self-esteem. Additionally, excursions, craft activities, discussions, and games were well received within the scope of the participants’ abilities.

However, some participants explicitly described an activity program as secondary to other factors, such as social interaction, rest, and care, in terms of well-being.

Many participants described the atmosphere at the day hospice as family-like, with mutual understanding being as important as the stimulating and invigorating effect of the community. Illness became the norm because *“…we don’t need pity*,* we need encouragement*,* and that’s just so different”* (9).

Quote (9): *“…that it does me a lot of good to be with people who are in a similar situation*,* because then you don’t see it as such a big deal anymore. Or you can talk to people who are affected in a different way than you can with family members or anyone else…you get to know new people who you get along with really well*,* and you feel like you’re in good hands here…. So I feel very comfortable here.”*

Additionally, participants emphasized the benefits of concrete organizational tips/experiences from others in dealing with the disease, perceiving them as valuable and, in some cases, reassuring.

### Care and emotional support from staff

Many participants found that having someone available at all times to discuss their concerns or address medical and organizational questions is reassuring. The feeling of warmth and human closeness or the sense of security provided by professionally competent staff was the most important factor.

Quote (12): *“…that all the employees are so attentive to me*,* that gives me … what you might call incredible support…. these efforts to help me as a person mean a lot to me. The care.”*

The staff pay attention to the wishes and needs of each patient and manage to cater for everyone. Some participants described their visit to the day hospice as almost luxurious due to this comprehensive care, as they were “pampered” by the staff. Some participants reported that this aspect of attentive support and comprehensive care by the staff was the most important factor when explaining the positive impact of the day hospice on their quality of life.

Quote (14): *“…I have a quality of life here that I can enjoy…. And that’s a wonderful feeling*,* yes….”*

For some participants, particularly those who required care at home or who had no relatives, the aspect of being looked after (meals and care) was at the top of their list of priorities. Additionally, participants perceived having everyday tasks that would otherwise have to be done at home taken care of for them at the day hospice as a relief because *“…I have someone where I am looked after…. There is no one at home…”* (8).

### Place of peace and relaxation

A feeling of “time out” is also a factor in improving quality of life. Some participants perceived the day hospice as a place of rest and relaxation in terms of the physical environment and human component. At the day hospice, they could take a break, relax, and leave many of their worries behind. Some participants appreciated the distance from their everyday lives at home because the day hospice is *“…such a safe space”* (5).

### Relief

Participants mentioned organizational relief as another factor in improving their quality of life. This included medical services that could sometimes avoid long journeys to different medical offices because *“you don’t have to run around everywhere*,* you don’t need appointments*,* … nothing”* (10). Additionally, the organization of nonmedical services, such as taxi rides, was greatly appreciated.

Except for one participant, all participants perceived the visit to the day hospice as a relief for their relatives. Reported reasons included relief from caregiving responsibilities on visiting days and a reduction in worries, as relatives knew their loved ones were in good hands and were happy that the patients themselves could enjoy new experiences.

Quote (8): *“Because they just have a little free time.”*

Quote (19): *“She [= the daughter] is very enthusiastic about it because she says*,* ‘Mom*,* you just have more involvement*,* more people to talk to*,* so you can talk about something other than us for a change.’ So she’s happy too.”*

Furthermore, relieving the burden on relatives is a source of reassurance and relief for most participants.

Quote (14): *“And I mean*,* of course I’m gone for a whole day. And that means that both my husband and my daughter can schedule appointments…. And that’s a kind of liberation*,* of course. In any case*,* it’s a relief*,* yes.”*

Since visit costs are covered by health insurance companies, the stay at the day hospice does not affect the financial situation of the participants or their relatives.

### Change in interaction with relatives

Most participants reported that visiting the day hospice had no effect on their communication with their relatives. Many participants reported saying little about their concerns to those around them, not wanting to burden anyone more than necessary, and wanting to focus on the aspect of “life.” Some participants reported that the exchange within the group increased their self-confidence in admitting their needs to others, making it easier for them to deal with these needs.

### Change in independence in the home environment

Many participants reported that visiting the day hospice did not result in any change in independence in their home environment. Most participants tried to maintain their daily routines as much as possible despite their illness. Some participants reported that staying at the day hospice led to increased stamina and a greater sense of security. Patients receiving parenteral nutrition at the day hospice reported being able to cope with their daily activities.

### Change in attitude toward life

Many participants did not see any fundamental change in their attitude toward life or outlook as a result of visiting the day hospice. Some patients reported that contact with the subject of dying (the death of other guests) enabled them to face or accept their own death in a more relaxed manner (less fear, more focus on normality and positive living), *“…you realize that you are ill. And that you will not live forever.”* (4).

Some participants gained a different perspective on their own suffering when confronted with the severity of their fellow patients’ illnesses, because *“…when I come back from the day hospice*,* I realize how good I actually have it compared to the many other patients I’ve met. And that motivates me too.”* (18).

### Change in meaning in life

Most participants reported that they had not experienced a profound change or found more meaning in life. Some participants stated that visiting the day hospice had helped them worry less about tomorrow and planning ahead.

Quote (4): *“…I say I live from day to day. That’s it. You always make the best of it. Just saying.”*

Participants emphasized that the day hospice had a positive influence on their zest for life, and they were grateful for every day. With the help of the day hospice, they did not give up, lived more freely from day to day, appreciated their time more, and did not want to waste it on things they did not consider “meaningful” for themselves.

Quote (14): *“Yes. So now I try to get up every day and live in such a way that I laugh every day*,* that I can really have fun every day and also talk about serious topics*,* that my life makes sense on that day. And I have to say*,* that wasn’t the case before… then I see in the day hospice how valuable this time is and how valuable laughter is*,* how valuable conversations are*,* how valuable people are … And I am extremely grateful for that….”*

### Support in determining the last will and testament

Most participants had already made arrangements regarding living wills or powers of attorney before visiting the day hospice or had done so independently of it. However, the possibility of support was met with broad approval and was considered an important aspect of a day hospice.

## Discussion

In Germany, day hospices are still a relatively new form of outpatient palliative care [2, 3]. Several day hospices have already been established and put into operation over the past three years. However, comprehensive coverage is not yet available in Germany [5].

Health insurance companies have specific requirements for establishing a day hospice [17]. Additionally, the German Hospice and Palliative Care Association (DHPV) [17] and Hannover Medical School [18] have published initial recommendations on the operation of day hospices. Day hospices remain an active area of research. Several studies have focused on the necessity and design of day hospices [3, 5, 6, 19]. However, little attention has been paid to practical everyday experiences. Conversely, this project, as part of the IMPULS study [12], aimed to shed light on the experiences of palliative care patients who have already visited or are still actively visiting a day hospice to take these experiences into account in the further development and establishment of day hospices.

Previous studies have highlighted the problem of social isolation among palliative care patients, with cancer patients accounting for the largest proportion of this patient group [20, 21]. The interview participants acknowledged the problem of social isolation and perceived the concept of day hospice as a possible solution or improvement. Additionally, participation in social life, experiences, adventures, and new interpersonal contacts are considered by project participants to be factors that contribute to an improvement in their quality of life. These findings are consistent with those from other countries, such as Austria, where the day hospice concept has been used for some time now [4, 8]. Therefore, the “day hospice” service offers a potential counterpoint to the isolation experienced by patients, which arises because those around them are often uncertain about these exceptional situations and may distance themselves from them [22, 23].

The provision of adventure therapy [24, 25] may be an important aspect of the day hospice concept for patients. Therefore, this service should be given greater consideration in the future development of day hospices. In this study, the participants perceived the community as a critical factor in improving their quality of life. Most respondents showed that daily life among like-minded or similarly affected people contributes to improving their quality of life, as they once again find life to be beautiful. These findings are consistent with those of day hospices in other countries [3, 8], such as Austria [4]. Improving quality of life through care, emotional support, and attention from trained personnel was particularly emphasized and valued [3]. However, the community of seriously ill people does not necessarily offer such clear added value for all patients, as some patients find it difficult to be confronted with the suffering of others.

In addition to the social component, medical care is also crucial, whether provided by specially trained nursing staff (e.g., wound dressings, port needle changes, parenteral nutrition, and pain pump monitoring) or by palliative care physicians through regular visits [3]. Although medical services provided by nursing staff are generally valued, regular physician visits are offered to varying degrees in day hospices [7]. The interview results showed that, in addition to the range of services offered by a day hospice, regular palliative medical physician services would be desirable. However, this depends on the day hospice location and the availability of palliative care physicians. As already described in publications [26], integrating the day hospice into existing medical networks can be highly beneficial in this regard, particularly since legal support for network structures is currently possible [27].

The approval process of some health insurance companies for day hospice stays was criticized, as it can cause considerable uncertainty among patients and is perceived as highly stressful. Therefore, transparent approval procedures similar to those established for specialized outpatient palliative care would be very helpful.

One key aspect highlighted by the interview participants was the relief it provided for family members. This result is consistent with the preliminary investigation results [7, 28]. Given that relatives and their health play a crucial role in the holistic care of seriously ill people, this aspect should be considered in the further development of day hospices.

Overall, the participants perceived the day hospice as a place of peace and relaxation where they could regain strength and confidence and rediscover meaning and joy in their lives. Therefore, day hospices are considered a fundamentally valuable component of outpatient palliative care. The study’s findings indicate that the hopes and expectations for this form of palliative care, which is still relatively new in Germany, can be fulfilled. Further development of this supply structure is needed.

## Limitations

This study has some limitations. This study was limited to guests of a day hospice with a medical and social focus located in a rural area. The opinions of guests from urban or differently structured day hospices may vary. All participants had a malignant tumor. The inclusion of patients with nonmalignant diseases could result in different response patterns and findings. Similarly, the integration of the day hospice into an established oncology/palliative care network might have affected patients’ experiences compared with other day hospices without such a connection. It cannot be ruled out that some of the interview questions were unconsciously perceived as suggestive by the participants. This could have unintentionally influenced their responses and should be taken into account when interpreting the results.

## Conclusions

The impact of day hospices extends beyond the visit and continues throughout the week. Social factors are more important than medical factors, and the attentive, family-like care provided by the staff is crucial. The program and activities improve patients’ quality of life. However, they are less important than social contacts or community. Visiting a day hospice can improve mental well-being by compensating for reduced social contact, reducing loneliness, providing relieving conversations with staff, and offering a feeling of security, distraction, and fun. Meeting people in similar situations has a positive effect, creating a sense of community, normality, and mutual understanding. Reducing the burden on relatives contributes to patients’ mental well-being.

## Supplementary Information

Supplementary Material 1.

## Data Availability

All relevant data are within the paper. The raw data analyzed in this study are available upon reasoned request to the corresponding author.

## Abbreviations

AAPV: General outpatient palliative care
SAPV: Specialized outpatient palliative care
IMPULS: Investigation into the implementation, realization, and benefits of a day hospice
DHPV: German Hospice and Palliative Care Association
