# Supplementary material for: Influence of a day hospice on the quality of life of palliative care patients: an interview-based qualitative survey
Source: BMC Palliat Care. 2026 Mar 11;25:92. doi: 10.1186/s12904-026-02052-w (PMC13063626; doi:10.1186/s12904-026-02052-w)
Supplement: Supplementary file 1 — Supplementary Material 1. [file 12904_2026_2052_MOESM1_ESM.docx]

**Appendix**

**Table 1** Interview guide used.

| **1.** | **Introduction** |
| --- | --- |
| 1.1. | Who are you? (Age, marital status, occupation, and interests) |
| 1.2. | What does a typical day look like for you? |
| **2.** | **Day Hospice: Evaluation** |
| 2.1. | Daily routines and initial expectations before the first visit |
| 2.1.1. | What did patients hope to gain from a day hospice? (For themselves, for their relatives, and in general) |
| 2.1.2. | What was your first impression? |
| 2.1.3. | What was your typical daily routine before becoming a guest at the day hospice? |
| 2.1.3.1. | Before the illness? |
| 2.1.3.2. | After the diagnosis or just before the first visit to the day hospice? |
| 2.2. | Impact of the visit on life |
| 2.2.1. | Would the quality of life be different in general if the day hospice did not exist? |
| 2.2.1.1. | If increased by visiting the day hospice, how do the interviewees determine this? How is this expressed? What is better? |
| 2.2.1.2. | If not increased by visiting the day hospice, why? What prevents a better quality of life? |
| 2.2.2. | Did the day hospice lead to more social contacts? |
| 2.2.2.1. | If so, why? |
| 2.2.2.2. | If not, why not? Do you not want any more social contacts? What prevents you from having more social contacts? |
| 2.2.3. | Has the day hospice led to more enjoyment of social life? |
| 2.2.3.1. | If so, how is this expressed? |
| 2.2.3.2. | If not, why not? Is this intentional? |
| 2.2.4. | Could the relatives be relieved by staying at the day hospice? |
| 2.2.4.1. | If so, how exactly? What does the relief consist of? |
| 2.2.4.2. | If not, why not? What prevents relief? |
| 2.2.5. | Has the stay at the day hospice changed the financial situation of the interviewees and/or their families? To what extent? (e.g., family caregivers can return to work) |
| 2.2.6. | Has staying at the day hospice changed the medical and/or palliative care provided? |
| 2.2.6.1. | If so, how exactly? |
| 2.2.6.2. | If not, why not? |
| 2.2.7. | Has the motivation to undergo hematological and/or oncological therapies changed as a result of staying at the day hospice? |
| 2.2.7.1. | If so, why and what exactly? |
| 2.2.7.2. | If not, why not? |
| 2.2.8. | Has the nutritional status (e.g., weight, food intake, and motivation to eat) changed as a result of staying at the day hospice? |
| 2.2.8.1. | If so, why and what exactly? |
| 2.2.8.2. | If not, why not? |
| 2.2.9. | Has the time (length, quality) that guests can spend in their home environment changed/increased as a result of the day hospice? |
| 2.2.9.1. | If so, why and how exactly? |
| 2.2.9.2. | If not, why not? |
| 2.2.10. | Has the day hospice changed the way you deal with family problems that may have arisen as a result of the illness? |
| 2.2.10.1. | If so, which ones and how exactly? |
| 2.2.10.2. | If not, why not? (Were there any at all?) |
| 2.2.11. | Has your independence and/or self-confidence outside the day hospice improved as a result of your stay there? |
| 2.2.11.1. | If so, why and how exactly? |
| 2.2.11.2. | If not, what are the reasons? |
| 2.2.12. | Has the day hospice changed your attitude toward life/death/illness or dying? |
| 2.2.12.1. | If so, why and how exactly? |
| 2.2.12.2. | If not, why not? |
| 2.2.13. | Has staying at the day hospice helped you find new/more meaning in life? |
| 2.2.13.1. | If so, to what extent? |
| 2.2.13.2. | If not, why not? |
| 2.2.14. | Was the day hospice able to provide support in discussing/planning/determining the last will and testament (e.g., patient decree, healthcare proxy, etc.)? |
| 2.2.14.1. | If so, how exactly? (e.g., in the sense that the conversation partner and/or family have addressed the issue, made arrangements, etc.) |
| 2.2.14.2. | If not, why not? |
| 2.2.14.3. | Is this even known/desired? |
| 2.2.15. | Could anything be changed at the day hospice to further improve/increase your quality of life during your visit? |
| 2.2.15.1. | If so, to what extent? What exactly? |
| 2.2.15.2. | If not, why not? |
| **3.** | **Closure** |
| 3.1. | Wishes for the future |
| 3.1.1. | Is there anything else you would like to tell us about day hospices? |
| 3.1.2. | Is there anything missing that we have not discussed? |
| 3.1.3. | Are there any other wishes for the future? |
